# Comments on “Until when will we issue specialist titles for physicians without medical residency?”

**DOI:** 10.1590/0100-6991e-20243828-en

**Published:** 2024-11-26

**Authors:** CÉSAR EDUARDO FERNANDES

**Affiliations:** 1 - Faculdade de Medicina do ABC (FMABC), Ginecologia - Santo André - SP - Brasil; 2 - Associação Médica Brasileira (AMB) - São Paulo - SP - Brasil

Upon receiving the invitation from the Editor-in-Chief of the Journal of the Brazilian College of Surgeons to the section Comments on the letter sent to the Editor by Professor Francisco Arsego de Oliveira, I would like, initially, to thank the writer for the endorsement given to our article “Why should Medical Societies increasingly take care of their Specialist Title exams and why should medical professionals obtain it?”^1^. In addition, I also want to highlight the comments added by the professor that greatly reinforce our arguments in defense of the zeal that everyone, especially the AMB, together with its specialty societies, should have with the specialist title (ST) and the need to make specialists aware so that they obtain it, regardless of whether they have already received their Specialist Qualification Registration (SQR) based solely on their certificate of completion of a medical residency program (MRP) recognized by the Ministry of Education (MEC). It is worth considering that the Regional Medical Councils (CRM) only comply with what the law prescribes, and it is not up to them, according to the current legislation, to conduct any merit analysis of the MRP graduates for the granting of the SQR.

I should note that, unlike what the legislation allows in our country, that is, obtaining the SQR issued by the CRM only with the presentation of the certificate of completion of the MRP, other countries have a policy for issuing the ST quite different from ours. They do consider medical residency as a necessary and indispensable prerequisite for the candidate to register and take the exams to obtain the ST. Without medical residency, it is not even allowed to apply for the exams. These are conducted through cognitive and practical evaluations conducted by a panel of independent experts for the effective proof of competencies, skills, and attitudes, after which the ST is granted to the approved candidate. Never, however, do they admit the issuance of the ST based exclusively on the certificate of completion of medical residency. This is indeed a necessary condition, but by no means sufficient for the granting of the ST. 

A good example is the one practiced by the USA. In that country, to be granted the ST, it is necessary to obtain a medical degree from a qualified medical school, complete three to five years of full-time experience in a residency program accredited by the Accreditation Council for Graduate Medical Education (ACGME), have an unrestricted medical license to practice in the United States or Canada, and pass exams designed and administered by the Board of Members of the American Board of Medical Specialties (ABMS)^2^. I should emphasize that in the USA medical residency is absolutely necessary, but never, in any way and under any circumstances, sufficient for the issuance of the ST.

In this context, it is worth repeating that the considered article^1^ pointed out that “The specialist title test plays a vital role in promoting excellence and protecting patient health while contributing to the continued advancement of the medical specialty. These exams should be designed to ensure high standards of competence and professionalism among physicians seeking to become specialists in a particular specialty”. This statement holds all the essence that guides the examination to obtain the ST granted by the AMB together with each of its 54 specialty societies. 

Thus, we believe that similar to what countries practice with specialist certification systems built and perfected over many decades, we must also evolve with our specialist recognition system in Brazil, with a view to the safety of the population that seeks competent, ethical, problem-solving, and safe care. The granting of the ST, as with any other norm that regulates the practice of medicine, must have patient safety as a non-negotiable premise. No other issue, of any other nature, should compromise this cornerstone in the ST granting, or patients could misleadingly think that they are being treated by specialists who have the necessary training and skills to do so. This is worrying.

With these considerations, we have the rational framework to answer the question that lends title to the aforementioned letter to the editor - Until when will we grant specialist titles to physicians without medical residency? All those involved with medical education, especially those concerned with the issuance of the specialist title, know that since its creation a few decades ago, the ST issued by the AMB in conjunction with the specialty societies allowed in its inception the enrollment of doctors without medical residency to apply.

Naturally, the purpose at that moment, decades ago, was a provisional one, to give the opportunity for many experienced doctors of the time, who had not done medical residency, and based on solid proven experience, to make their specialty official in case of approval in the then held tests. Furthermore, it is worth remembering that although medical residency began in Brazil during the forties of the last century, programs were scarce and only a small portion of medical school graduates sought this type of training. As a result, there was a small number of physicians with complete residency during the first decades following the creation of these programs. This situation without any standardization lasted for a long time, and the regulation of medical residency only occurred in 1977 through the presidential decree, which defined that the MRP would constitute a modality of postgraduate education for physicians, in the form of a specialization course, characterized by in-service training, in a regime of exclusive dedication, working in health institutions, university or not, under the guidance of medical professionals with high ethical and professional qualifications^3^. After this period and encouraged by the regulation, MRPs increased in Brazil.

Given that small number of physicians with completed residency, it would not be advisable at the time to prevent all other doctors who did not meet this prerequisite from taking the ST test. It is clear, therefore, that this initial permission for doctors without medical residency to apply for the ST tests should be understood as transition rules and that, as soon as possible, they would be extinguished. 

Nowadays, that idea of allowing doctors without residency to take the ST exam no longer has any reason to exist. But the fact is that this permission still exists today. This situation can only change by legal provision. Many decades have passed, and we have MRP graduates in large numbers, supposedly with adequate training, complying with the competence matrices required for the adequate training of the medical specialist. We agree that this needs to change. We need to evolve with this issue with the same responsibility verified in countries that treat the training of medical specialists with the necessary seriousness. We need to also institute in Brazil the completion of a MRP as a mandatory prerequisite to the ST test. It is past time we conferred seriousness to our criteria for ST granting. The practice of medicine and the population require this change in criteria for the granting of ST, aiming at the practice of specialized medical care that is competent, effective, and safe.

To conclude, it is worth considering another enormous concern not accounted for in our legislation, but already crystallized in countries with more mature and improved evaluation methods. In our country, once the ST is issued, it is valid for a long time and does not oblige the specialists, at any time and for any reason, to prove that they are up to date and have incorporated, into their skills, abilities and attitudes, all the technical-scientific advances that have occurred over time in their specific area of expertise. 

Just to stay with the example of a country already mentioned here, I turn once again to the USA. There, the process in force requires a recertification every five years through accreditation programs. The process that is being currently implemented is called the ABMS program for Maintenance of Certification (MOC)^4^, and considers that, after the initial certification, doctors will be able to maintain their certification as specialists (ST) if they participate in a continuous certification process. In other words, proof of up-to-date knowledge is required for the maintenance of the ST. Not here. But this is another critical point that can and should be addressed in depth at another time. Let us stay, for now, with our proposition of the mandatory completion of a MRP as a mandatory prerequisite for applying to the ST exam. One step at a time but always with the greater objective of qualifying medical care in favor of patient safety.
